# Peripheral gene dysregulation in Negr1-deficient mice: insights into possible links with affective behavior

**DOI:** 10.3389/fnmol.2025.1602201

**Published:** 2025-07-08

**Authors:** Abdulkadir Yusif Maigoro, Jangrae Kim, Seoyeon Cho, Ara Yoo, Soojin Lee

**Affiliations:** Department of Microbiology and Molecular Biology, Chungnam National University, Daejeon, Republic of Korea

**Keywords:** neuronal growth regulator 1, obesity, depression, differentially expressed genes, interleukin-17

## Abstract

**Introduction:**

Neuronal growth regulator 1 (NEGR1) is a brain-enriched membrane protein with mild expression in peripheral tissues such as adipose tissue and skeletal muscle. Genome-wide association studies have implicated NEGR1 as a risk factor for human diseases including obesity, autism, and depression, but its molecular function remains poorly understood.

**Methods:**

To explore NEGR1’s role in peripheral-to-brain communication, we conducted RNA-seq analysis on four peripheral tissues—intestine, skeletal muscle, liver, and epididymal white adipose tissue—collected from *Negr1* knockout mice. Differentially expressed genes (DEGs) were identified and subjected to Gene Ontology (GO) enrichment analyses.

**Results:**

The DEG analysis revealed dysregulation of ion channels and transporters, potentially contributing to AP-1-mediated inflammatory responses in peripheral tissues. Additionally, interleukin (IL)-17 signaling emerged as a key pathway that may mediate systemic inflammation in *Negr1*-deficient mice.

**Discussion:**

These findings suggest a novel role for NEGR1 in modulating peripheral inflammatory responses and support the hypothesis that peripheral immune dysregulation may contribute to depressive-like behaviors in *Negr1*-deficient mice. This work enhances our understanding of NEGR1’s function in peripheral tissues and its possible involvement in peripheral-central immune crosstalk relevant to psychiatric disorders.

## 1 Introduction

Major depressive disorders, schizophrenia, bipolar disorder, and autism are among the most common psychiatric disorders that are considerably expensive to treat (Uher and Zwicker, 2017). Although the etiology of mental illness has not been elucidated completely, it is possibly caused by several genetic and non-genetic factors. Common genetic factors have been implicated in the etiology of multiple mental disorders, including schizophrenia, autism spectrum disorder (ASD), anxiety, and major depression (Taylor et al., 2019). Furthermore, several psychiatric disorders are associated with an increased risk of neurodegenerative diseases, particularly Alzheimer’s disease (Martin-Sanchez et al., 2021).

Neuronal growth regulator 1 (NEGR1) is a glycosylphosphatidylinositol (GPI)-anchored cell adhesion molecule with three highly glycosylated C2-type immunoglobulin (Ig)-like domains (Kim et al., 2014). Similar to other GPI-anchored proteins, NEGR1 is a cell surface protein and is particularly enriched in the membrane raft fraction. NEGR1 is implicated in several neuronal functions such as synaptic plasticity, and neurite outgrowth (Noh et al., 2020).

Although *NEGR1* has been recognized as an obesity risk gene (Willer et al., 2009), recent large genome-wide association studies (GWAS) have identified *NEGR1* as one of the most significant risk genes for major depressive disorders (MDD) (Howard et al., 2018; Wray et al., 2018). We have previously demonstrated that Negr1 knockout (KO) mice exhibited depression- and anxiety-like behaviors (Noh et al., 2019). Furthermore, a series of GWAS have revealed NEGR1 as a causal risk factor for ASD (Grove et al., 2019), schizophrenia (Deng et al., 2025), and Alzheimer’s disease (Ni et al., 2018). Maccarrone et al. (2013) analyzed the cerebrospinal fluid protein expression patterns and reported NEGR1 as a highly reliable biomarker for MDD. Protein analysis of male patients with schizophrenia revealed elevated NEGR1 protein levels, suggesting that altered NEGR1 levels may serve as a biological risk marker for a wide spectrum of psychiatric conditions (Karis et al., 2018).

Although NEGR1 is highly expressed in the brain, a considerable amount of NEGR1 protein has been detected in peripheral tissues, including adipose tissues, heart, testes, and stomach (Joo et al., 2019). Furthermore, we previously reported the involvement of NEGR1 in intracellular lipid trafficking by interacting with the Niemann-Pick type C2 protein (Kim et al., 2017). Negr1-deficient mice display abnormalities in peripheral tissues such as increased adiposity, hepatic fat accumulation, and muscle atrophy (Joo et al., 2019). In addition, NEGR1 interacts with cluster of differentiation 36 (CD36), the main lipid transporter in different immune and non-immune cells (Yoo et al., 2022). Collectively, these results suggest that NEGR1 has distinct functions in non-CNS cells.

The brain is no longer considered the center of mental health; rather, it is believed that disturbances in the mind are intricately connected to the whole body (The Lancet Psychiatry, 2023). The brain dynamically processes body signals generated by the peripheral organs (The Lancet Psychiatry, 2023). Immune dysregulation, inflammation, and the endocrine system interact with both central and peripheral systems to worsen psychiatric conditions (Renoir et al., 2013). In addition, multiple studies have demonstrated the role of bidirectional communication in the gut-brain axis in several psychiatric diseases, including anxiety and depression (Clapp et al., 2017).

In this study, we hypothesized that the affective behavior observed in *Negr1^−/−^* mice is at least partly attributable to the abnormalities in the peripheral organs. We analyzed the expression profiles of four peripheral tissues, epididymal WAT (eWAT), liver, skeletal muscle, and intestine, to identify the biological pathways most dysregulated in *Negr1^−/−^* mice and to interpret their crosstalk between the brain and body. This study provides new insights into the cellular function of NEGR1 as well as peripheral factors that may contribute to mental illness.

## 2 Materials and methods

### 2.1 Mice and tissue collection for RNA sequencing (RNA-seq)

Wild-type (WT) and *Negr1^−/−^* C57BL/6 mice (Joo et al., 2019) were maintained under a 12:12 h light–dark cycle at 22 ± 1°C and 55% humidity. All animal experiments were approved by the Institutional Animal Care and Use Committee of Chungnam National University. Two WT and two *Negr1^−/−^* male mice (aged 10–12 weeks) were used to obtain four different tissues, eWAT, liver, intestinal epithelial cells (IEC), and gastrocnemius (GA) skeletal muscle tissue for the transcriptome analysis.

IEC were purified from dissected small intestines as previously described (Gracz et al., 2012). Briefly, after the mice were euthanized, an ileal segment approximately 20 cm proximal to the ileocecal valve was obtained. Next, the tissues were finely chopped and washed thoroughly with ice-cold phosphate-buffered saline (PBS). After the ileum segments were shaken gently in ice-cold PBS containing 5 mM EDTA and 5 mM DTT for 10 min, the remaining samples were incubated in a 37°C shaker with PBS containing 30 mM EDTA for 30 min. Afterward, the IEC were then centrifuged at 1,000×*g* for 10 min.

### 2.2 RNA extraction, quality assessment, and sequencing

Total RNA was extracted from tissue samples using the TRIzol reagent (Invitrogen, USA), and its quality was assessed using the Agilent 2,100 Bioanalyzer (Agilent Technologies, USA). Samples with an RNA integrity number (RIN) > 7.0 were retained for sequencing. RNA extraction, library preparation, and sequencing were performed by E-Biogen Inc. (South Korea).

RNA-seq libraries were constructed using the Lexogen QuantSeq 3′ mRNA-Seq Library Prep Kit (Lexogen, Austria) according to the manufacturer’s instructions. Sequencing was performed on an Illumina NextSeq 550 platform, generating single-end 75 bp reads. Each sample yielded approximately 19 million reads. FastQC (v0.11.9) was used for quality control, and no reads were flagged as low quality. Clean reads were aligned to the mouse reference genome (UCSC mm10/GRCm38) using Bowtie2 (v2.3.5). Gene-level read counts were normalized using the trimmed mean of M values (TMM) method and expressed as counts per million (CPM) with the EdgeR package (v3.28.1) in R (Robinson et al., 2010). The RNA-seq data presented in this publication have been deposited in NCBI’s Sequence Read Archive (https://www.ncbi.nlm.nih.gov/sra) under the project accession number PRJNA1281825. Additional data are provided within the manuscript and Supplementary materials.

### 2.3 Identification of differentially expressed genes (DEGs)

Differentially expressed genes (DEGs) were identified using both the DESeq2 (v1.34.0) and limma packages in R (version 4.2.2). Raw RNA-seq data were obtained from four mice—two wild-type and two Negr1 knockout animals—across four different tissues. The sequencing reads were aligned and quantified using CLC Genomics Workbench (v11.0, Qiagen, Germany). Gene expression was calculated as fragments per kilobase of transcript per million mapped reads (FPKM). For statistical analysis, raw count data were used as input for DESeq2, which performed normalization and differential expression testing. The results were cross-validated using limma to enhance robustness.

Given the limited number of biological replicates (n = 2 per group per tissue), conventional statistical thresholds (e.g., adjusted *p*-value < 0.05) would result in low statistical power and a high risk of false negatives. To address this limitation and support hypothesis generation, we adopted a relaxed DEG selection strategy based on a fold-change threshold (|log2FC| > 1) and a minimum expression cutoff, without strict p-value filtering. Specifically, genes with an average raw count of ≥ 10 in at least one group (KO or WT) were included in the analysis, to reduce noise from low-abundance transcripts. Although nominal *p*-values were recorded, we prioritized effect size and biological plausibility over statistical significance. This approach aligns with previously published methods for exploratory transcriptomic analyses under small-sample conditions (Subramanian et al., 2005; Timmons et al., 2015).

### 2.4 Gene ontology (GO) functional enrichment analysis

Gene Ontology (GO) and Kyoto Encyclopedia of Genes and Genomes (KEGG) pathway enrichment analyses were performed using the Database for Annotation, Visualization and Integrated Discovery (DAVID, https://david.ncifcrf.gov/, version 6.8) to investigate the biological characteristics of the selected DEGs in *Negr1^−/−^* mice. The pathways were visualized in the form of a bubble plot using bioinformatics tools. Statistical significance was set at *p* < 0.05. A Venn diagram was plotted using an online tool (https://bioinfogp.cnb.csic.es/tools/venny/, version 2.1) to identify the overlapping genes among the DEGs from the four different tissues.

### 2.5 Protein–protein interaction (PPI) network construction and module analysis

Protein–protein interaction (PPI) networks were constructed using the Search Tool for the Retrieval of Interacting Genes (STRING) database (https://string-db.org/, version 11.5). PPIs with a combined score > 0.4 were considered significant. All results were visualized using the STRING plugin in the Cytoscape software (version 3.7.1). CytoHubba, a plugin for Cytoscape, was employed to identify the top 10 hub genes based on their connectivity within the gene interaction network, using the Degree algorithm to rank the nodes by the number of direct connections. The ClueGO Cytoscape plugin was used to visualize non-redundant biological terms in a functionally grouped network.

### 2.6 Heatmap analysis of IL-17 pathway genes

Gene expression values for IL-17-related genes were visualized using the pheatmap package in R (version 4.2.2; https://www.r-project.org). For each tissue, gene expression levels were compared between the WT and *Negr1*^−/−^ mice. A red-to-blue gradient was applied to indicate differential expression, representing up- and downregulation, respectively.

### 2.7 Determination of colon length, real-time PCR, and immunoblotting

To measure the length of the large intestine, male mice were sacrificed by cervical dislocation, and the entire colon was carefully excised up to the ileocecal junction. After rinsing with ice-cold PBS, the colon was gently blotted dry using filter paper, and its length was measured.

Quantitative real-time PCR (qRT-PCR) was performed using the SYBR Green Realtime PCR Master Mix (Toyobo, Japan) and the Bio-Rad CFX Connect™ Real-Time PCR Detection System (Bio-Rad Laboratories, USA).

Immunoblotting was conducted as previously described (Yoo et al., 2022). The following primary antibodies were used for protein detection: anti-IL-17 receptor A (Abcam, USA), anti-vinculin, and anti-NEGR1 (both from Santa Cruz Biotechnology, USA).

## 3 Results

### 3.1 Identification of DEGs from the four peripheral tissues

**Figure 1:**
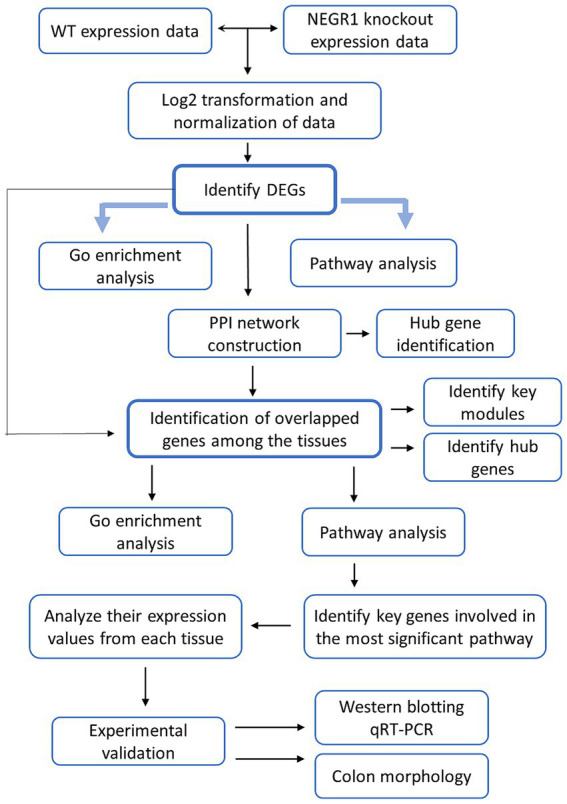
Description of the experimental design.

Transcriptome analysis was conducted using four different tissues-eWAT, liver, GA muscle, and intestine-in WT and *Negr1^−/−^* mice to analyze DEGs in Negr1-deficient mice (Figure 1). RNA-seq datasets were standardized and normalized, and *Negr1* expression levels in each tissue were confirmed (Supplementary Figure S1A). The distribution of DEGs in each tissue is illustrated using volcano plots in Supplementary Figure S1B. In intestinal tissue, *Scg5*, *Acsbg1*, and *Slfn1* were the most highly upregulated genes, while *Tomm6os*, *Bdnf*, and *Snord83b* were the most strongly downregulated. In the liver, *Hist1h4n*, *Hspa1a*, and *Rgs1* showed marked upregulation, whereas *Nnat*, *Peg3*, and *Gnao1* were significantly downregulated. In eWAT, *Pitx2*, *Slc22a1*, and *Pkhd1* were the most upregulated genes, while *Dmrtb1*, *Zfp454*, and *Ccdc169* showed notable downregulation. In GA muscle, *Prm1*, *Fscn2*, *F5* were the most upregulated, whereas *Plb1*, *Tnfrsf22*, and *Nudt15* were strongly downregulated. The gene names and the corresponding gene symbols are listed in Supplementary Table S1.

Due to the small sample size, applying strict significance thresholds (e.g., adjusted *p*-value < 0.05) identified only a limited number of DEGs. Therefore, we applied a more permissive DEG selection strategy using a fold-change cutoff (|log2FC| > 1) and a minimum expression threshold, without excluding genes based solely on *p*-values. For downstream functional analyses, only DEGs with a fold change ≥ 2 were retained to focus on robust expression changes.

The number of DEGs was the highest in eWAT among the four tissues, and 3,393 DEGs were found in eWAT, of which 2,177 were upregulated and 1,216 were downregulated. Furthermore, 2,672 DEGs were found in the liver tissue, of which 1,262 were upregulated and 1,410 were downregulated. A total number of 2,512 DEGs were found in the GA muscle tissue: 1,195 were upregulated and 1,317 were downregulated. Finally, 1,125 DEGs were found in intestine, of which 713 were upregulated and 412 were downregulated.

### 3.2 GO functional enrichment analysis

**Figure 2:**
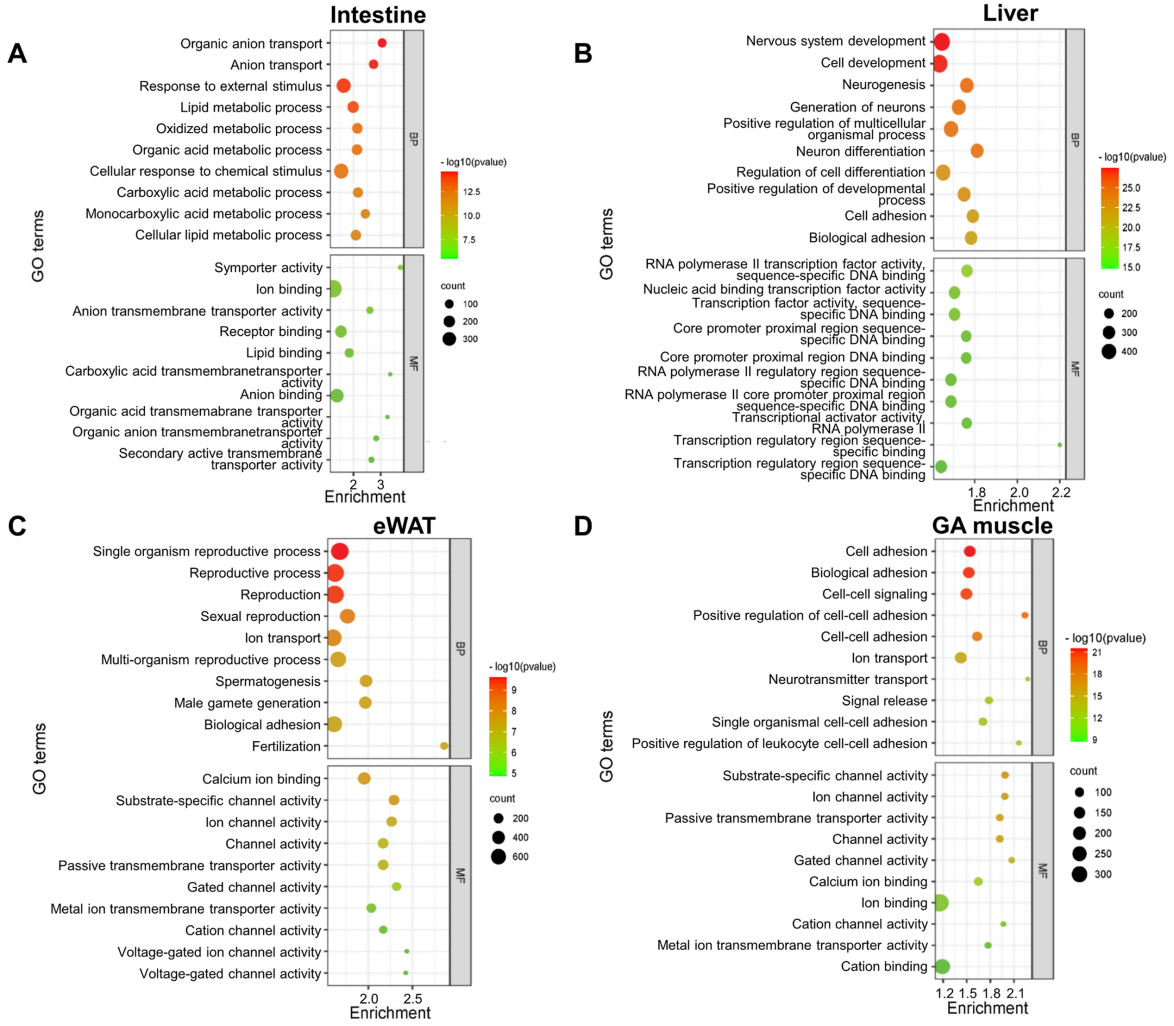
Bubble chart showing enriched Gene Ontology (GO) terms of differentially expressed genes (DEGs) between WT and *Negr1^−/−^* mice from four different tissues [**(A)** intestine, **(B)** liver, **(C)** eWAT, **(D)** GA muscle], constructed using a bioinformatics database. The enrichment factor represents the ratio of the observed frequency of DEGs associated with a GO term to the expected frequency based on the background gene distribution. Higher enrichment values indicate greater overrepresentation. The color and size of the dots represent the *p*-value range and the number of DEGs associated with each GO term, respectively. GO terms are arranged in order of statistical significance for both biological process (BP) and molecular function (MF).

We next wanted to obtain insight into the biological functions of NEGR1 in each tissue using the identified DEGs, for which GO terms and pathway enrichment analyses were performed using the DAVID software and arranged in the order of the most significant *p*-values (Figure 2). The GO analysis revealed that the biological processes (BP) of the intestine were enriched in organic anion transport, anion transport, response to external stimuli, and lipid metabolic processes. The liver is involved in nervous system development, cell development, generation of neurons, and neurogenesis. In eWAT, BP was enriched in single-organism reproductive processes, reproductive processes, and sexual reproduction. Muscle GO BP were enriched in cell adhesion, biological adhesion, and cell–cell signaling. The GO analysis results for the intestine showed molecular function (MF) enrichment in symporter activity, ion binding, receptor binding, and lipid binding. In addition, the MF analysis of the liver revealed significantly enriched DEGs in RNA polymerase II transcription factor activity, and nucleic acid-binding transcriptional factor activity. In eWAT, GO analysis targeting the MF was enriched for calcium ion binding, substrate-specific channel activity, and ion channel activity. Similarly, the GO for MF in the GA muscle was enriched in substrate-specific channel activity, ion channel activity, and passive transmembrane transporter activity.

### 3.3 KEGG pathway enrichment analysis and identification of hub genes

**Figure 3:**
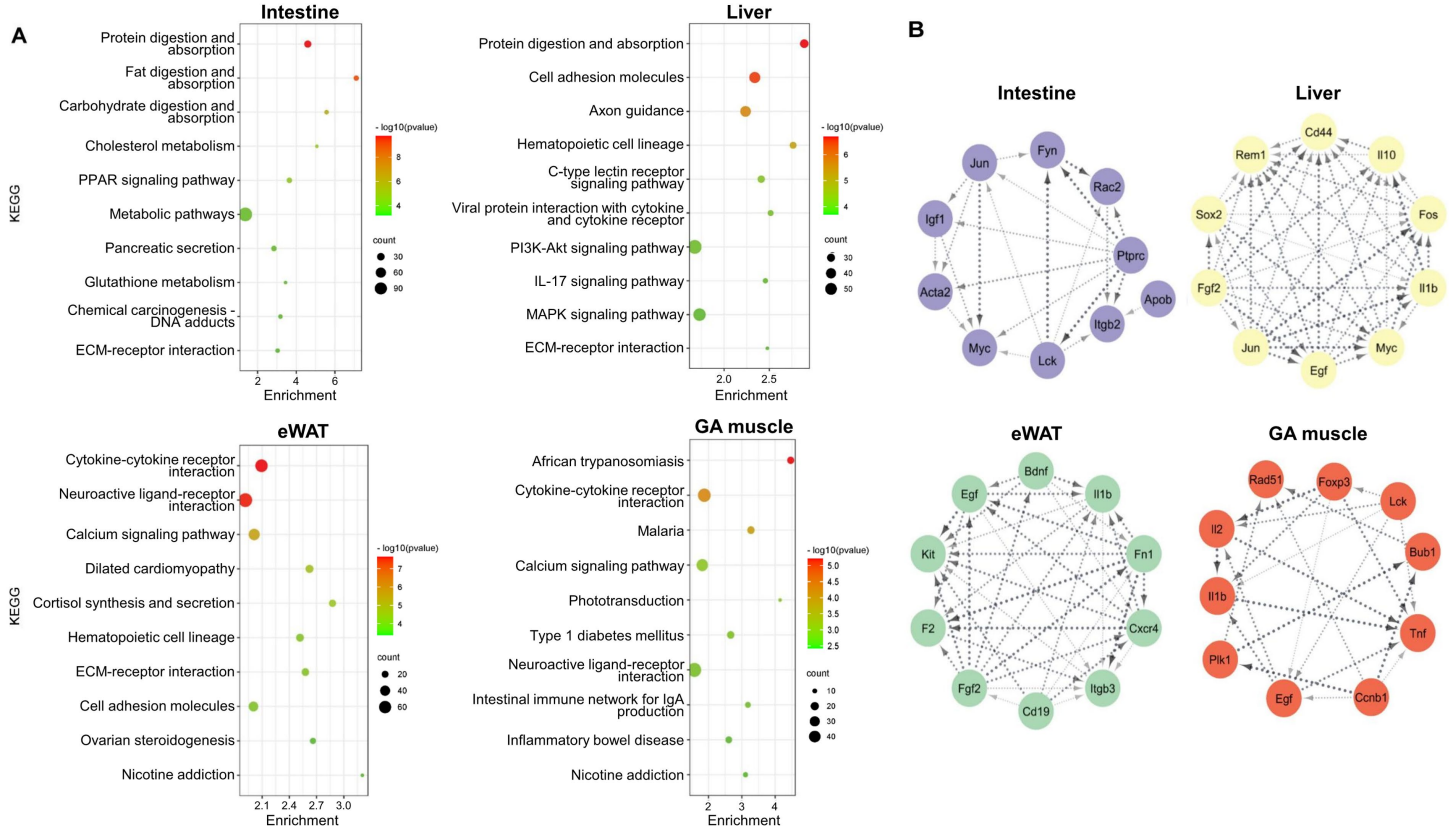
**(A)** Bubble chart showing enriched KEGG pathways of DEGs between WT and *Negr1^−/−^* mice from four different tissues (intestine, liver, eWAT, and GA muscle). The enrichment factor represents the ratio of the observed DEG frequency to the expected frequency based on background distribution. Higher enrichment values indicate greater overrepresentation. The color and size of the dots represent the range of the *p*-value and the number of the DEGs mapped to each KEGG pathway, respectively. KEGG pathways are arranged in order of statistical significance. **(B)** Analysis of the top 10 hub genes for the four different tissues. The CytoHubba plugin from Cytoscape was utilized to identify and visualize hub genes from our DEGs with ≥ two-fold change. Hub genes were analyzed based on their degree of connectivity.

The top pathways identified in the intestine, liver, eWAT, and GA muscle tissues were obtained through the KEGG analysis, as shown in Figure 3A. Intestinal DEGs were enriched in protein digestion and absorption, fat digestion and absorption, and carbohydrate digestion and absorption pathways. Protein digestion and absorption, cell adhesion molecules, and axon guidance were the most enriched pathways in the liver. In eWAT, DEGs were enriched in cytokine-cytokine receptor interactions, neuroactive ligand-receptor interactions, and calcium signaling pathways. The GA muscle DEGs were enriched in African trypanosomiasis and cytokine-cytokine receptor interactions.

We next constructed a PPI network for the respective DEGs using the STRING database. Topology results derived from CytoHubba revealed that protein tyrosine phosphatase receptor type c (*Ptprc*), *Jun*, and integrin subunit beta 2 (*Itgb2*) were the top three hub genes with the highest node degrees in the intestine (Figure 3B). *Jun*, epidermal growth factor (*Egf*), and *Myc* were the top three hub genes with higher node degrees for the liver tissue. Furthermore, the PPI network of eWAT revealed that fibronectin 1 (*Fn1*), *Egf*, and coagulation factor 2 (*F2*) were the top three hub genes with the highest node degrees. The top three most significant hub genes were tumor necrosis factor (*Tnf*), *Egf*, and interleukin 1 beta (*Il1b*) for the GA muscle tissue. Differential expression of selected hub genes with > 2-fold change from RNA-seq analysis was validated using qRT-PCR (Supplementary Figure S2A).

### 3.4 Identification of overlapping DEGs and functional analysis

**Figure 4:**
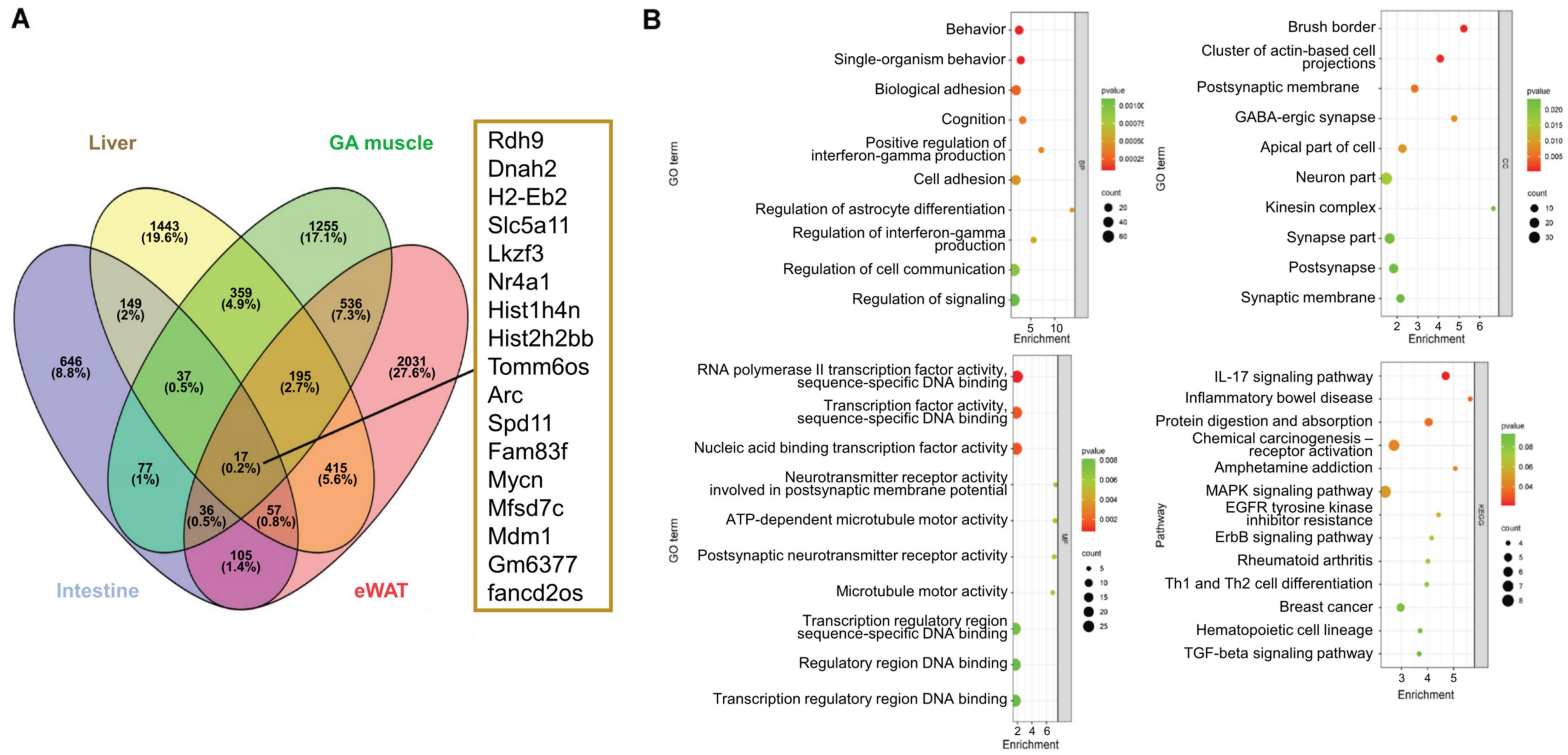
**(A)** A Venn-diagram demonstrating the overlapping genes among the four tissues and their respective DEGs. **(B)** The overlapping genes at least in three tissues were selected for the GO analysis and pathway analysis. The enrichment factor represents the ratio of the DEGs number to the total gene number.

To explore the fundamental role of NEGR1, we selected DEGs that were broadly dysregulated across the four tissues (Figure 4A). Only 0.2% of the total genes (17 genes) were identified as overlapping DEGs in the four tissues of Negr1 KO mice. We next selected DEGs that overlapped in at least three of the four tissues (342 DEGs) and further analyzed their functions and pathways.

As shown in Figure 4B, the GO enrichment analysis of these overlapping genes identified terms with *p*-values ≤ 0.002 for BP, ≤ 0.006 for MF, and ≤ 0.02 for cellular component (CC). The KEGG pathway enrichment results revealed *p*-values ranging from 0.08 to 0.04. The GO BP of these overlapping genes demonstrated that BP was enriched in behavior and single-organism behavior. The CC was enriched in the brush border, a cluster of actin-based cell projections, and GABAergic synapses. The MF of the overlapped genes was enriched in RNA polymerase II transcription factor activity, sequence-specific DNA binding, and neurotransmitter receptor activity. The most enriched KEGG pathways included the IL-17 signaling pathway, inflammatory bowel disease, protein digestion and absorption, and MAPK signaling pathway.

### 3.5 ClueGO enrichment analysis

**Figure 5:**
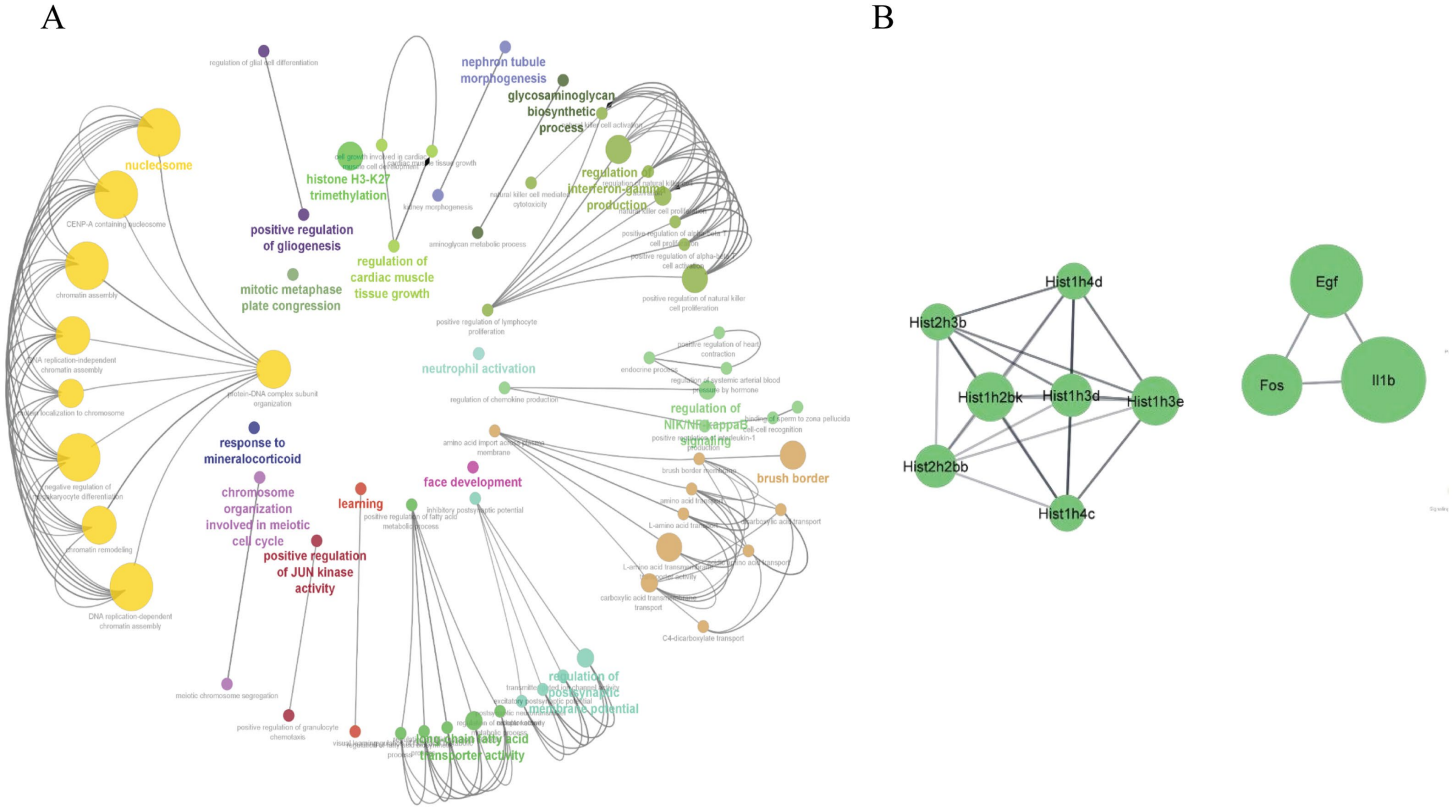
**(A)** The ClueGO plugin from Cytoscape was used to analyze and visualize GO enrichment for the overlapped genes derived from the four different tissues (intestine, liver, eWAT, and GA muscle). Important biological processes (BP), cellular components (CC), and molecular functions (MF) involved in the DEGs are shown. **(B)** The top 10 hub genes were identified from the PPI network of the overlapped genes using CytoHubba, a plugin in Cytoscape. The degree of connectivity increased with an increase in the node size.

The Cytoscape plugin ClueGO was used to study the functional enrichment of the identified overlapping genes from DEGs datasets of the intestine, liver, eWAT, and GA muscle tissues. ClueGO was used to visualize the GO terms of the identified PPI complex network of overlapping genes. The MF, CC, and BP terms of GO functional enrichment analysis are shown in Figure 5A. These were enriched in the positive regulation of the JUN kinase activity (GO:0043507), positive regulation of gliogenesis (GO:0042065), brush border (GO:0005903), and learning (GO:0007612). Furthermore, the Cytoscape plugin Cytohubba was used to generate the top 10 hub genes, including *Hist1h3d*, *Egf*, *Fos*, and *Il1b*, using the PPI network of overlapping DEGs (Figure 5B).

### 3.6 Analysis of IL-17 pathway components

**Figure 6:**
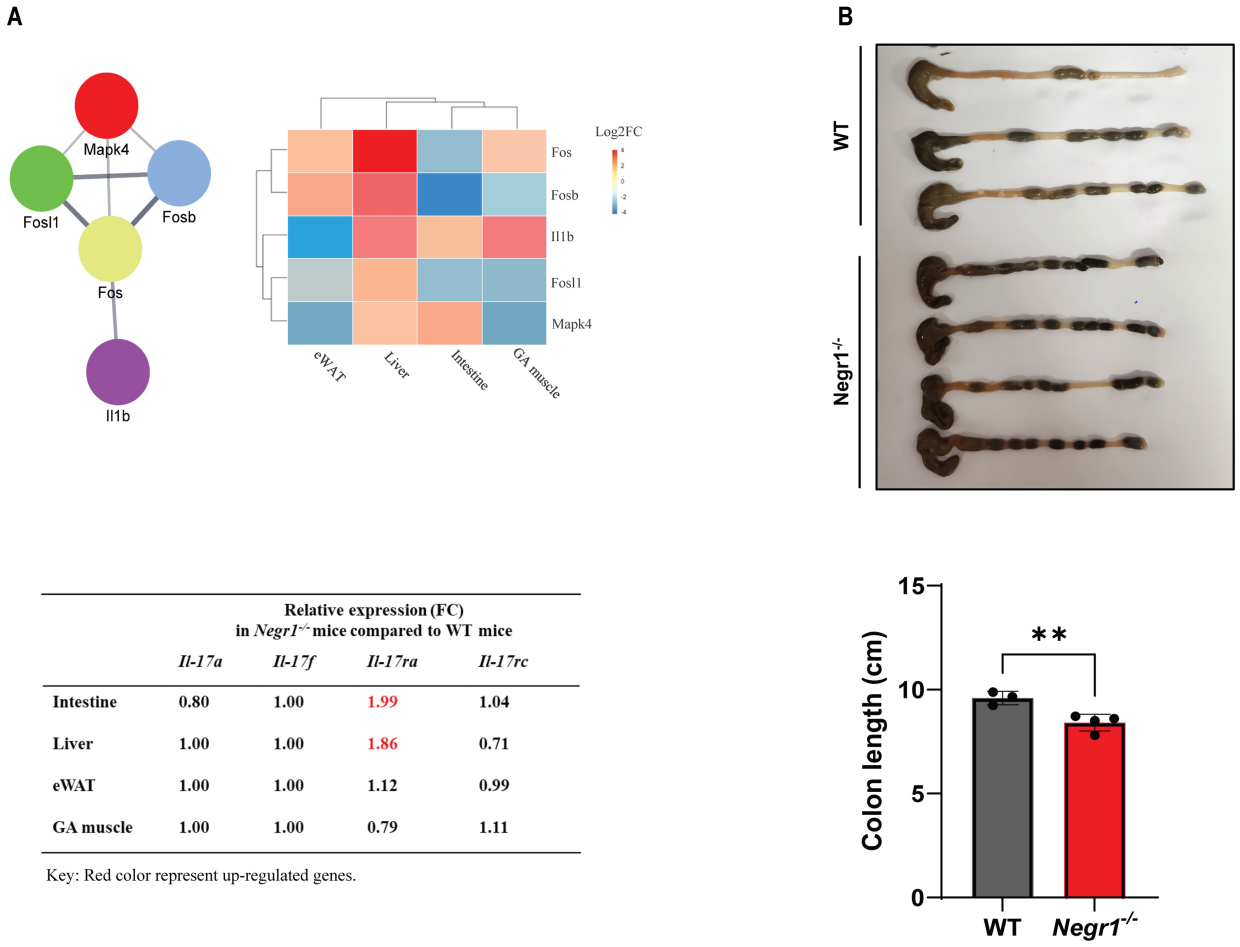
**(A)** The STRING plugin in Cytoscape was used to create a PPI network of the five IL-17 pathway-related genes. Gene expression across the four tissues was visualized using pheatmap. A red-to-blue gradient was applied to represent upregulation and downregulation, respectively. The table presents the relative expression levels of major IL-17 ligands and receptors in *Negr1^−/−^* mice compared to that in WT mice. The relative expression levels of *Il17ra* in the intestine and liver is highlighted in red to indicate their potential biological relevance. **(B)** Measurement of colon length dissected from 5-week-old male WT and *Negr1^−/−^* mice.

The KEGG analysis of overlapped gene sets demonstrated that the IL-17 pathway and inflammatory bowel disease were the most affected by Negr1 deficiency in multiple tissues (Figure 4B). Among the genes involved in the IL-17 pathway, *Fos*, *Fosb*, FOS-like antigen 1 *(Fosl1)*, *Il1b*, and mitogen-activated protein kinase 4 (*Mapk4*) were identified as highly dysregulated genes. Individual expression profiles of these DEGs were analyzed and compared for each tissue (Figure 6A). These five genes were either up- or downregulated in all four tissues (> two-fold). In addition, we found that the mRNA expression of major IL-17 ligands and IL-17 receptor C was not changed significantly, except that the expression of IL-17 receptor A (*Il17ra*) was substantially increased in the intestine and liver of *Negr1^−/−^* mice than in WT mice. To validate this at the protein level, we performed western blotting using ileal tissue from the small intestine and observed approximately 1.7-fold increase in IL-17RA protein expression in *Negr1^−/−^* mice compared to WT mice (Supplementary Figure S2C).

### 3.7 Measurement of colon length

The colon length is commonly used to assess the severity of inflammatory bowel disease. The entire colon was dissected from 5-week-old male *Negr1^−/−^* and WT mice, and its length was measured. The colon length of *Negr1^−/−^* mice was significantly shorter than that of WT mice (Figure 6B), measuring approximately 87% of the WT colon length. In addition, a more severe decrease was observed when the colon length of 16-week-old mice was measured (Supplementary Figure S3). The mean length of *Negr1^−/−^* mice was 6.62 ± 0.54 cm, which was 61% of WT mice, demonstrating that Negr1 deficiency induced chronic intestinal inflammation.

## 4 Discussion

NEGR1 is associated with several human pathologies, including cancer, obesity, diabetes, neurodevelopmental disorders, depression, and ASD (Kim et al., 2014; Szczurkowska et al., 2018; Noh et al., 2020). We investigated the DEGs of four different tissues (intestine, liver, eWAT, and GA muscle) of *Negr1^−/−^* mice, as well as the overlapping DEGs throughout these peripheral tissues to interpret a possible link with different parts of the body underlying these human diseases. In addition, we performed enrichment analysis using DAVID to investigate the involvement of DEGs from each tissue in BP, MF, and KEGG pathways and determine their functional annotations.

GO BP were highly enriched for “nervous system development” in the liver and “cell–cell adhesion” in the GA muscle (Figures 2B,D), reflecting the known function of NEGR1 as a cell adhesion molecule in the brain that is involved in adult neurogenesis (Noh et al., 2019). These findings, along with the GO BP results for overlapping genes that indicated “behavior” and “cognition” (Figure 4B), demonstrated that *Negr1* deficiency-driven DEGs in peripheral tissues may affect brain function.

The GO MF across all four tissues and GO BP of the intestine (Figure 2A) consistently indicated that membrane transport activity for ions or lipids was the most affected in *Negr1^−/−^* mice. This aligns with previous findings that NEGR1 interacts with the Na^+^/K^+^-ATPase beta1 subunit (Cheon et al., 2021) and a major fatty acid transporter, CD36 (Yoo et al., 2022). The enriched KEGG pathway in both the intestine and liver was “protein digestion and absorption” (Figure 3A), suggesting the involvement of NEGR1 in the extracellular cleavage of large molecules and transport of their digested nutrients, such as sugar, peptides, and fatty acids, into the cell. Because different types of ion channels and transporters are localized to cholesterol-enriched lipid raft microdomains (Dart, 2010), and NEGR1 is exclusively found in this region (Kim et al., 2014), our data suggest that NEGR1 is associated with several transport machinery located in membrane rafts.

The GO CC analysis of the overlapping DEGs further revealed the functional association of NEGR1 with nutrient transporters (Figure 4B), with “brush border” ranked the highest. The brush border comprises microvilli and a plasma membrane that supports the microfilaments. This aids in providing efficient digestion and absorption of nutrients as well as protecting the intestinal mucosa. Brush border components are essential partners of the gut microbiota and are efficient signaling platforms for physiological stimuli (Delacour et al., 2016). This highlights the function of NEGR1 in intestinal integrity as well as in the gut microbiome. Considering that GO BP is enriched in “behavior” from the common DEGs (Figure 4A), the gut-brain axis could be associated with the anxio-depressive behaviors of *Negr1*^*−*/−^ mice. Gut microbiome dysbiosis has been observed in Negr1-deficient mice (M. Kim, Chungnam National University, unpublished data, 2024), which could have been caused by an altered intestinal host environment.

The PPI network analysis of each tissue was conducted to identify the top 10 hub genes (Figure 3B). The majority of the top 10 hub genes, including *Ptprc*, *Tnf*, *Il1b*, and *Itgb2*, are strongly associated with inflammation and the immune system (Li et al., 2022). Furthermore, the PPI analysis of the overlapping genes identified *Fos*, *Egf*, and *Il1b* as crucial hub genes (Figure 5B). EGF and IL-1 were recognized to regulate the expression of genes involved in inflammation and cancer (Kasza, 2013). Jun and Fos are the major components of the activator protein 1 (AP-1) transcription factor and are crucial regulator of inflammation (Atsaves et al., 2019). In addition, TNF-mediated AP-1 activation promotes the expression of proinflammatory genes (Kyriakis, 1999). The enrichment of GO MF of the overlapped genes in “RNA polymerase II transcription factor activity, sequence-specific DNA binding” (Figure 4B) and the above findings demonstrate that AP-1 activity and immune system are highly affected in the peripheral tissues of Negr1 KO mice.

IL-17 is a highly versatile cytokine that promotes protective innate immunity against pathogens and contributes to the development of inflammatory diseases (Zenobia and Hajishengallis, 2015). JunB, an AP-1 component, is essential for Th17 cell development, whereas IL-17 significantly induces AP-1 promoter activity (Kim et al., 2013; Yamazaki et al., 2017). In addition, “cytokine-cytokine receptor interaction” was selected by the GO KEGG analyses in both eWAT and GA muscle (Figure 3A), and “IL-17 signaling pathway” was selected from GO KEGG of the overlapping genes (Figure 4B). The expression of genes involved in IL-17 activation, as well as IL-17 receptor A (*Il17ra*), was dysregulated in many tissues (Figure 6A).

Because IL-17 can affect CNS-resident cells, such as astrocytes and neurons, IL-17 has been implicated in several diseases, including inflammatory, autoimmune, and neurological disorders (Kolbinger et al., 2016). IL-17A released from different immune cells may be responsible for the development of neuropsychiatric symptoms associated with depression (Nadeem et al., 2017). IL-17A causes depression-like symptoms by increasing the NF-κB and p38MAPK signaling in different brain regions in mice. In addition, elevated IL-17A levels in the blood correlate with phenotypic severity in individuals with ASD (Wong and Hoeffer, 2018). Administration of IL-17A to the fetal brain promotes an ASD-like phenotype in mice by altering fetal brain development (Choi et al., 2016).

Depression and obesity are highly comorbid and frequently co-occur with inflammation mediated by proinflammatory cytokines such as IL-6 and TNF-α (Ouakinin et al., 2018). In this vicious cycle, IL-17 plays an important role in neuroimmune interactions (Lu et al., 2023). The IL-17 signaling system is not restricted to a particular tissue; it operates in several other tissues, such as the bone, articular cartilage, brain, lung, kidney, and intestine (Moseley et al., 2003). Considering the proposed linking function of IL-17 between depression, obesity, and inflammation and that IL-17 signaling was highly enriched in the common DEGs of Negr1-deficient mice, we propose that IL-17 as a crucial component of inter-organ communication and responsible for the affective behavior in *Negr1^−/−^* mice.

In addition to the IL-17 signaling pathway, “inflammatory bowel disease” and “protein digestion and absorption” were identified as highly significant KEGG pathways from the overlapping genes (Figure 4B). IL-17 plays a pivotal role in the pathogenesis of inflammatory bowel disease (Schmitt et al., 2021). Examination of the colon length revealed a chronic inflammation in the intestine of Negr1 KO mice (Figure 6B and Supplementary Figure S3), demonstrating impaired intestinal homeostasis in Negr1-deficient mice.

A major limitation of this study is the small number of biological replicates per group, which inherently reduces the statistical power of differential expression analyses. In small datasets, even genes with large fold-changes may not meet conventional significance thresholds, increasing the risk of false negatives. To mitigate this, we employed a permissive DEG selection strategy, prioritizing genes with substantial fold-change (|log2FC| > 1) and sufficient expression, rather than relying strictly on *p*-value cutoffs. This approach aligns with prior work in exploratory transcriptomics under constrained conditions (Ioannidis, 2005; Subramanian et al., 2005; Timmons et al., 2015), and enabled the identification of biologically plausible candidates for further validation.

Despite the relaxed threshold, several key findings were independently validated. The abrogated expression of Negr1 was confirmed, and RNA-seq results for multiple hub genes were supported by qRT-PCR. Additionally, the increased expression of Il-17RA detected by RNA-seq was validated at the protein level by Western blotting. Functional enrichment analyses also revealed pathways consistent with known roles of NEGR1 in metabolic and immune processes. Collectively, these findings support the suitability of our DEG selection approach for identifying meaningful transcriptional alterations in Negr1-deficient mice.

In this study, analyses of the widely dysregulated genes across peripheral tissues of *Negr1^−/−^* mice, along with GO data from individual tissues, indicate that the Negr1-deficiency-driven behavioral change could be related to a dysregulation of nutrient and ion transporters (also shown as “brush border”) and subsequent AP-1-involved inflammatory responses of peripheral tissues. In addition, IL-17 could be mediating the systematic inflammatory response in *Negr1^−/−^* mice. Altogether, this study may contribute to understanding the function of peripheral cells in neuropsychiatric disorders and elucidating the crosstalk between the brain and periphery.

## Data Availability

TThe RNA-seq data presented in this publication have been deposited in NCBI’s Sequence Read Archive (https://www.ncbi.nlm.nih.gov/sra) under the project accession number PRJNA1281825. Additional data are provided within the article/Supplementary materials. Further imquiries can be directed to the corresponding author.

## Supplementary material

Supplementary Table 1 List of genes and their descriptions.

Supplementary Table 2 Genes showing more than 2-fold change.

Supplementary Data Sheet 1 Volcano plots for differentially expressed genes in four tissues.

Supplementary Data Sheet 2 Validation of DEGs by quantitative RT-PCR.

Supplementary Data Sheet 3 Colon length measurements of 16-week-old mice.

## References

[ref1] Atsaves V. Leventaki V. Rassidakis G. Z. Claret F. X. (2019). AP-1 transcription factors as regulators of immune responses in cancer. Cancers 11:1037. doi: 10.3390/cancers11071037, PMID: 31340499 PMC6678392

[ref2] Cheon Y. Yoo A. Seo H. Yun S.-Y. Lee H. Lim H.. (2021). Na/K-ATPase beta1-subunit associates with neuronal growth regulator 1 (NEGR1) to participate in intercellular interactions. BMB Rep. 54:164. doi: 10.5483/BMBRep.2021.54.3.116, PMID: 32958118 PMC8016658

[ref3] Choi G. B. Yim Y. S. Wong H. Kim S. Kim H. Kim S. V.. (2016). The maternal interleukin-17a pathway in mice promotes autism-like phenotypes in offspring. Science 351, 933–939. doi: 10.1126/science.aad0314, PMID: 26822608 PMC4782964

[ref4] Clapp M. Aurora N. Herrera L. Bhatia M. Wilen E. Wakefield S. (2017). Gut microbiota's effect on mental health: the gut-brain axis. Clin Pract 7:987. doi: 10.4081/cp.2017.987, PMID: 29071061 PMC5641835

[ref5] Dart C. (2010). Lipid microdomains and the regulation of ion channel function. J. Physiol. 588, 3169–3178. doi: 10.1113/jphysiol.2010.191585, PMID: 20519314 PMC2976012

[ref6] Delacour D. Salomon J. Robine S. Louvard D. hepatology (2016). Plasticity of the brush border—the yin and yang of intestinal homeostasis. Nat. Rev. Gastroenterol. Hepatol. 13, 161–174. doi: 10.1038/nrgastro.2016.5, PMID: 26837713

[ref7] Deng M. G. Wang K. Liu F. Zhou X. Nie J. Q. Zhao Z. H.. (2025). Shared genetic architecture and causal relationship between frailty and schizophrenia. Schizophrenia (Heidelb) 11:24. doi: 10.1038/s41537-024-00550-5, PMID: 39984493 PMC11845589

[ref8] Gracz A. D. Puthoff B. J. Magness S. T. (2012). Identification, isolation, and culture of intestinal epithelial stem cells from murine intestine. Methods Mol. Biol. 879, 89–107. doi: 10.1007/978-1-61779-815-3_6, PMID: 22610555 PMC6218244

[ref9] Grove J. Ripke S. Als T. D. Mattheisen M. Walters R. K. Won H.. (2019). Identification of common genetic risk variants for autism spectrum disorder. Nat. Genet. 51, 431–444. doi: 10.1038/s41588-019-0344-8, PMID: 30804558 PMC6454898

[ref10] Howard D. M. Adams M. J. Shirali M. Clarke T. K. Marioni R. E. Davies G.. (2018). Genome-wide association study of depression phenotypes in UK biobank identifies variants in excitatory synaptic pathways. Nat. Commun. 9:1470. doi: 10.1038/s41467-018-03819-3, PMID: 29662059 PMC5902628

[ref11] Ioannidis J. P. (2005). Why most published research findings are false. PLoS Med. 2:e124. doi: 10.1371/journal.pmed.0020124, PMID: 16060722 PMC1182327

[ref12] Joo Y. Kim H. Lee S. Lee S. (2019). Neuronal growth regulator 1-deficient mice show increased adiposity and decreased muscle mass. Int. J. Obes. 43, 1769–1782. doi: 10.1038/s41366-019-0376-2, PMID: 31086253

[ref13] Karis K. Eskla K. L. Kaare M. Taht K. Tuusov J. Visnapuu T.. (2018). Altered expression profile of IgLON family of neural cell adhesion molecules in the dorsolateral prefrontal cortex of schizophrenic patients. Front. Mol. Neurosci. 11:8. doi: 10.3389/fnmol.2018.00008, PMID: 29434535 PMC5797424

[ref14] Kasza A. (2013). IL-1 and EGF regulate expression of genes important in inflammation and cancer. Cytokine 62, 22–33. doi: 10.1016/j.cyto.2013.02.007, PMID: 23481102

[ref15] Kim H. Chun Y. Che L. Kim J. Lee S. Lee S. (2017). The new obesity-associated protein, neuronal growth regulator 1 (NEGR1), is implicated in Niemann-pick disease type C (NPC2)-mediated cholesterol trafficking. Biochem. Biophys. Res. Commun. 482, 1367–1374. doi: 10.1016/j.bbrc.2016.12.043, PMID: 27940359

[ref16] Kim H. Hwang J. S. Lee B. Hong J. Lee S. (2014). Newly identified Cancer-associated role of human neuronal growth regulator 1 (NEGR1). J. Cancer 5, 598–608. doi: 10.7150/jca.8052, PMID: 25057311 PMC4107236

[ref17] Kim G. Khanal P. Lim S. C. Yun H. J. Ahn S. G. Ki S. H.. (2013). Interleukin-17 induces AP-1 activity and cellular transformation via upregulation of tumor progression locus 2 activity. Carcinogenesis 34, 341–350. doi: 10.1093/carcin/bgs342, PMID: 23125217

[ref18] Kolbinger F. Huppertz C. Mir A. Di Padova F. (2016). IL-17A and multiple sclerosis: signaling pathways, producing cells and target cells in the central nervous system. Curr. Drug Targets 17, 1882–1893. 26953244 10.2174/1389450117666160307144027

[ref19] Kyriakis J. M. (1999). Activation of the AP-1 transcription factor by inflammatory cytokines of the TNF family. Gene Expr. 7:217–231. 10440223 PMC6174675

[ref20] Li G. Sun J. Zhang J. Lv Y. Liu D. Zhu X.. (2022). Identification of inflammation-related biomarkers in diabetes of the exocrine pancreas with the use of weighted gene co-expression network analysis. Front. Endocrinol. (Lausanne) 13:839865. doi: 10.3389/fendo.2022.839865, PMID: 35498402 PMC9046596

[ref21] Lu Y. Zhang P. Xu F. Zheng Y. Zhao H. (2023). Advances in the study of IL-17 in neurological diseases and mental disorders. Front. Neurol. 14:1284304. doi: 10.3389/fneur.2023.1284304, PMID: 38046578 PMC10690603

[ref22] Maccarrone G. Ditzen C. Yassouridis A. Rewerts C. Uhr M. Uhlen M.. (2013). Psychiatric patient stratification using biosignatures based on cerebrospinal fluid protein expression clusters. J. Psychiatr. Res. 47, 1572–1580. doi: 10.1016/j.jpsychires.2013.07.021, PMID: 23962679

[ref23] Martin-Sanchez A. Pinero J. Nonell L. Arnal M. Ribe E. M. Nevado-Holgado A.. (2021). Comorbidity between Alzheimer's disease and major depression: a behavioural and transcriptomic characterization study in mice. Alzheimers Res. Ther. 13:73. doi: 10.1186/s13195-021-00810-x, PMID: 33795014 PMC8017643

[ref24] Moseley T. Haudenschild D. Rose L. Reddi A. H. (2003). Interleukin-17 family and IL-17 receptors. Cytokine Growth Factor Rev. 14, 155–174. doi: 10.1016/s1359-6101(03)00002-9 12651226

[ref25] Nadeem A. Ahmad S. F. Al-Harbi N. O. Fardan A. S. El-Sherbeeny A. M. Ibrahim K. E.. (2017). IL-17A causes depression-like symptoms via NFκB and p38MAPK signaling pathways in mice: Implications for psoriasis associated depression. Cytokine 97, 14–24. doi: 10.1016/j.cyto.2017.05.018, PMID: 28570931

[ref26] Ni H. Xu M. Zhan G. L. Fan Y. Zhou H. Jiang H. Y.. (2018). The GWAS risk genes for depression may be actively involved in Alzheimer's disease. J. Alzheimers Dis. 64, 1149–1161. doi: 10.3233/JAD-180276, PMID: 30010129

[ref27] Noh K. Lee H. Choi T.-Y. Joo Y. Kim S.-J. Kim H.. (2019). Negr1 controls adult hippocampal neurogenesis and affective behaviors. Mol. Psychiatry 24, 1189–1205. doi: 10.1038/s41380-018-0347-3, PMID: 30651602

[ref28] Noh K. Park J. C. Han J. S. Lee S. J. (2020). From bound cells comes a sound mind: The role of neuronal growth regulator 1 in psychiatric disorders. Exp. Neurobiol. 29, 1–10. doi: 10.5607/en.2020.29.1.1, PMID: 32122104 PMC7075657

[ref29] Ouakinin S. R. Barreira D. P. Gois C. (2018). Depression and obesity: integrating the role of stress, neuroendocrine dysfunction and inflammatory pathways. Front. Endocrinol. 9:431. doi: 10.3389/fendo.2018.00431, PMID: 30108549 PMC6079193

[ref30] Renoir T. Hasebe K. Gray L. (2013). Mind and body: how the health of the body impacts on neuropsychiatry. Front. Pharmacol. 4:158. doi: 10.3389/fphar.2013.00158, PMID: 24385966 PMC3866391

[ref31] Robinson M. D. McCarthy D. J. Smyth G. K. (2010). edgeR: a Bioconductor package for differential expression analysis of digital gene expression data. Bioinformatics 26, 139–140. doi: 10.1093/bioinformatics/btp616, PMID: 19910308 PMC2796818

[ref32] Schmitt H. Neurath M. F. Atreya R. (2021). Role of the IL23/IL17 pathway in Crohn’s disease. Front. Immunol. 12:622934. doi: 10.3389/fimmu.2021.622934, PMID: 33859636 PMC8042267

[ref33] Subramanian A. Tamayo P. Mootha V. K. Mukherjee S. Ebert B. L. Gillette M. A.. (2005). Gene set enrichment analysis: a knowledge-based approach for interpreting genome-wide expression profiles. Proc. Natl. Acad. Sci. USA 102, 15545–15550. doi: 10.1073/pnas.0506580102, PMID: 16199517 PMC1239896

[ref34] Szczurkowska J. Pischedda F. Pinto B. Manago F. Haas C. A. Summa M.. (2018). NEGR1 and FGFR2 cooperatively regulate cortical development and core behaviours related to autism disorders in mice. Brain 141, 2772–2794. doi: 10.1093/brain/awy190, PMID: 30059965 PMC6113639

[ref35] Taylor M. J. Martin J. Lu Y. Brikell I. Lundstrom S. Larsson H.. (2019). Association of Genetic Risk Factors for psychiatric disorders and traits of these disorders in a Swedish population twin sample. JAMA Psychiatry 76, 280–289. doi: 10.1001/jamapsychiatry.2018.3652, PMID: 30566181 PMC6439816

[ref36] The Lancet Psychiatry (2023). Centring the periphery. Lancet Psychiatry 10:1. doi: 10.1016/S2215-0366(22)00408-4, PMID: 36526342

[ref37] Timmons J. A. Szkop K. J. Gallagher I. J. (2015). Multiple sources of bias confound functional enrichment analysis of global -omics data. Genome Biol. 16:186. doi: 10.1186/s13059-015-0761-7, PMID: 26346307 PMC4561415

[ref38] Uher R. Zwicker A. (2017). Etiology in psychiatry: embracing the reality of poly-gene-environmental causation of mental illness. World Psychiatry 16, 121–129. doi: 10.1002/wps.20436, PMID: 28498595 PMC5428165

[ref39] Willer C. J. Speliotes E. K. Loos R. J. Li S. Lindgren C. M. Heid I. M.. (2009). Six new loci associated with body mass index highlight a neuronal influence on body weight regulation. Nat. Genet. 41, 25–34. doi: 10.1038/ng.287, PMID: 19079261 PMC2695662

[ref40] Wong H. Hoeffer C. (2018). Maternal IL-17A in autism. Exp. Neurol. 299, 228–240. doi: 10.1016/j.expneurol.2017.04.010 28455196 PMC5656543

[ref41] Wray N. R. Ripke S. Mattheisen M. Trzaskowski M. Byrne E. M. Abdellaoui A.. (2018). Genome-wide association analyses identify 44 risk variants and refine the genetic architecture of major depression. Nat. Genet. 50, 668–681. doi: 10.1038/s41588-018-0090-3, PMID: 29700475 PMC5934326

[ref42] Yamazaki S. Tanaka Y. Araki H. Kohda A. Sanematsu F. Arasaki T.. (2017). The AP-1 transcription factor JunB is required for Th17 cell differentiation. Sci. Rep. 7:17402. doi: 10.1038/s41598-017-17597-3, PMID: 29234109 PMC5727176

[ref43] Yoo A. Joo Y. Cheon Y. Lee S. J. Lee S. (2022). Neuronal growth regulator 1 promotes adipocyte lipid trafficking via interaction with CD36. J. Lipid Res. 63:100221. doi: 10.1016/j.jlr.2022.100221, PMID: 35526561 PMC9189132

[ref44] Zenobia C. Hajishengallis G. J. V. (2015). *Porphyromonas gingivalis* virulence factors involved in subversion of leukocytes and microbial dysbiosis. Virulence 6, 236–243. doi: 10.1080/21505594.2014.999567, PMID: 25654623 PMC4601496

