# Supplementary material for: Peripheral gene dysregulation in Negr1-deficient mice: insights into possible links with affective behavior
Source: Front Mol Neurosci. 2025 Jul 8;18:1602201. doi: 10.3389/fnmol.2025.1602201 (PMC12279845; doi:10.3389/fnmol.2025.1602201)
Supplement: Supplementary Table 1 — List of genes and their descriptions. [file Table_1.docx]

Supplementary Material

**Supplementary Table S1**. List of genes and their descriptions

| Gene symbol | Description |
| --- | --- |
| *Negr1*  *Il-17*  *Il-17ra*  *Il-17rc*  *Ptprc*  *Jun*  *Itgb2*  *Lck*  *Egf*  *Myc*  *Cd44*  *Rem1*  *Fn1*  *F2*  *Bdnf*  *Kit*  *Il1b*  *Foxp3*  *Plk1*  *Bub1*  *Fos*  *Fosb*  *Fosl1*  *Mapk4*  *Tnf*  *Cd36*  *Scg5*  *Acsbg1*  *Slfn1*  *Cym*  *Bambi-ps1*  *Tomm6os*  *Snord83b*  *Efnb3*  *Nr4a1*  *Hist1h4n*  *Hspa1a*  *Rgs1*  *Ccrn4l*  *Nnat*  *Peg3*  *Gnao1*  *Arntl*  *Dmrtb1*  *Zfp454*  *Ccdc169*  *Rtp3*  *Pitx2*  *Slc22a1*  *Pkhd1*  *Folr1*  *Daw1*  *Plb1*  *Tnfrsf22*  *Nudt15*  *Zfp341*  *Prm1*  *Fscn2*  *F5*  *Card11*  *Slc35d2* | Neuronal growth regulator 1  Interleukin 17  Interleukin17 receptor A  Interleukin17 receptor C  Phosphatase receptor type c  Jun proto-oncogene  Integrin subunit beta 2  Lymphocyte protein tyrosine kinase  Epidermal growth factor  Myelocytomatosis oncogene  Cd44 antigen  Rad and gem related GTP binding protein 1  Fibronectin 1  Coagulating factor 2  Brain derived neurotrophic factor  Kit proto-oncogene receptor tyrosine kinase  Interleukin 1 beta  Forkhead box P3  Polo like kinase 1  Bub1, mitotic checkpoint serine/threonine kinase  Fos proto-oncogene  FBJ murine osteosarcoma viral oncogene homolog B  Fos-like antigen 1  Mitogen-activated protein kinase 4  Tumor necrosis factor  Cluster of differentiation 36  Neuroendocrine protein 7B2  Acyl-CoA synthetase, Bubblegum Family, member 1  Schlafen family member 1  Chymosin  BMP and activing membrane-bound inhibitor, pseudogene  Translocase of outer mitochondrial membrane 6, opposite strand  Small nucleolar RNA, C/D box 83B  Ephrin B3  Nuclear receptor subfamily 4, groug A, member 1  H4 clustered histone 18  Heat shock protein 1A  Regulator of G-protein signaling 1  Nocturnin  Neuronatin  Paternally expressed 3  Guanine nucleotide binding protein, alpha O  Basic helix-loop-helix ARNT like 1  DMRT-like family B with proline-rich C-terminal, 1  Zinc finger protein 454  Coiled-coil domain containing 169  Receptor transporter protein 3  Paired-like homeodomain transcription factor 2  Solute carrier family 22, member 1  Polycystic kidney and hepatic disease 1  Folate receptor alpha  Dynein assembly factor with WDR repeat domains 1  Phospholipase B1  Tumor necrosis factor receptor superfamily, member 22  Nudix hydrolase 15  Zinc finger protein 341  Protamine 1  Fascin actin-bundling protein 2  Coagulation factor V  Caspase recruitment domain family, member 11  Solute carrier family 35, member D2 |
