# Supplementary material for: Peripheral gene dysregulation in Negr1-deficient mice: insights into possible links with affective behavior
Source: Front Mol Neurosci. 2025 Jul 8;18:1602201. doi: 10.3389/fnmol.2025.1602201 (PMC12279845; doi:10.3389/fnmol.2025.1602201)
Supplement: Supplementary Data Sheet 1 — Volcano plots for differentially expressed genes in four tissues. [file Data_Sheet_1.pdf]

Supplementary Material

A

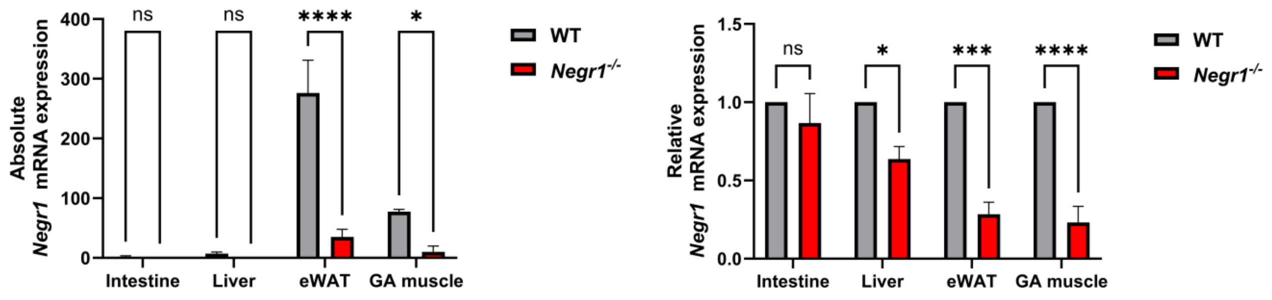

B

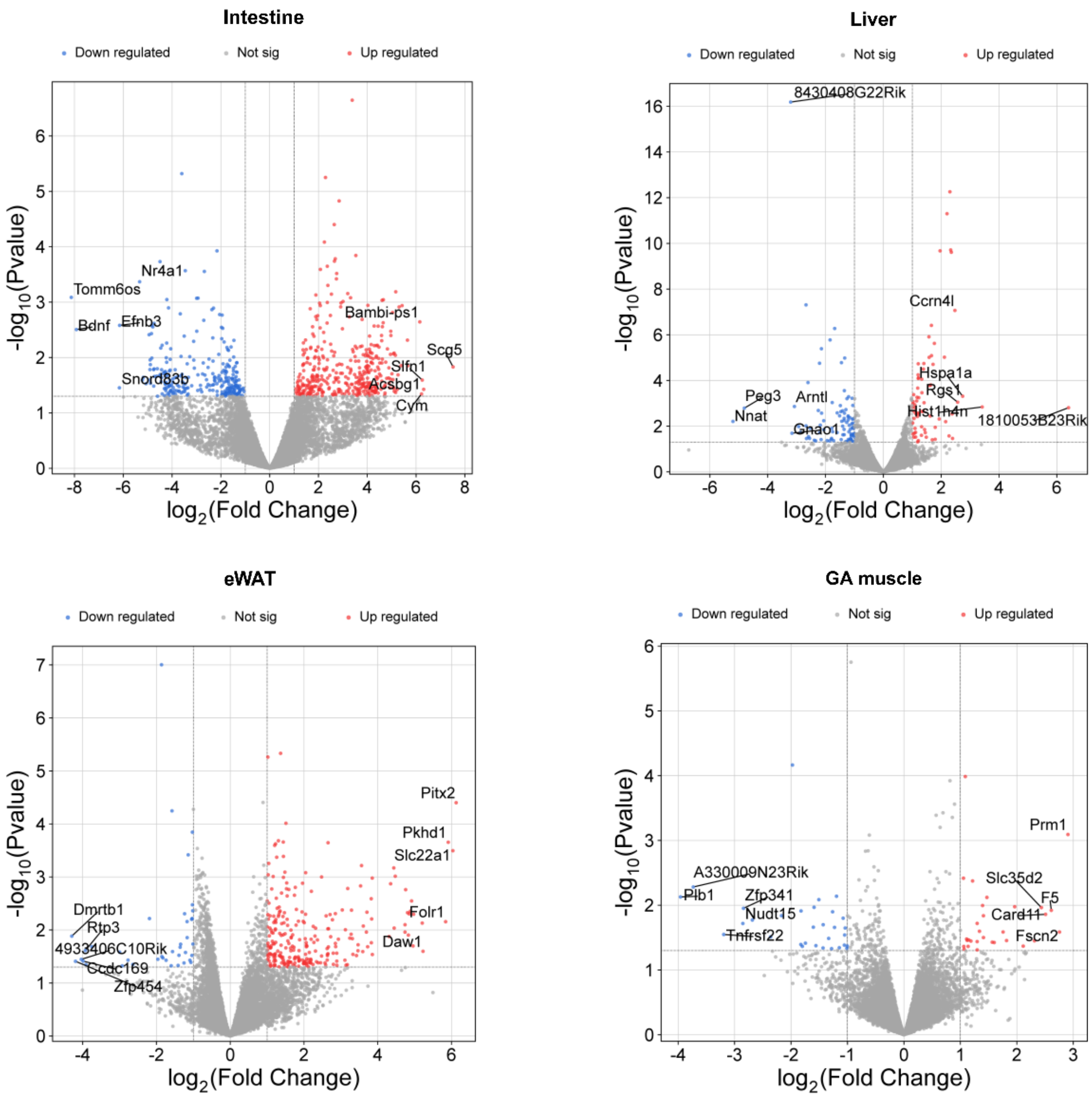

Supplementary Figure S1. (A) Expression levels of Negr1 in RNA-seq data from WT and *Negr1*$^{-/-}$ mice across four tissues: intestine, liver, epididymal white adipose tissue (eWAT), and gastrocnemius (GA) muscle. (B) Volcano plots showing differentially expressed genes (DEGs) identified by differential expression analysis comparing WT and *Negr1*$^{-/-}$ mice in each tissue. Red dots represent significantly upregulated genes, blue dots indicate significantly downregulated genes, and gray dots denote non-significant or non-differentially expressed genes (based on the specified p-value threshold).
