# Supplementary material for: Peripheral gene dysregulation in Negr1-deficient mice: insights into possible links with affective behavior
Source: Front Mol Neurosci. 2025 Jul 8;18:1602201. doi: 10.3389/fnmol.2025.1602201 (PMC12279845; doi:10.3389/fnmol.2025.1602201)
Supplement: Supplementary Data Sheet 2 — Validation of DEGs by quantitative RT-PCR. [file Data_Sheet_2.pdf]

Supplementary Material

A

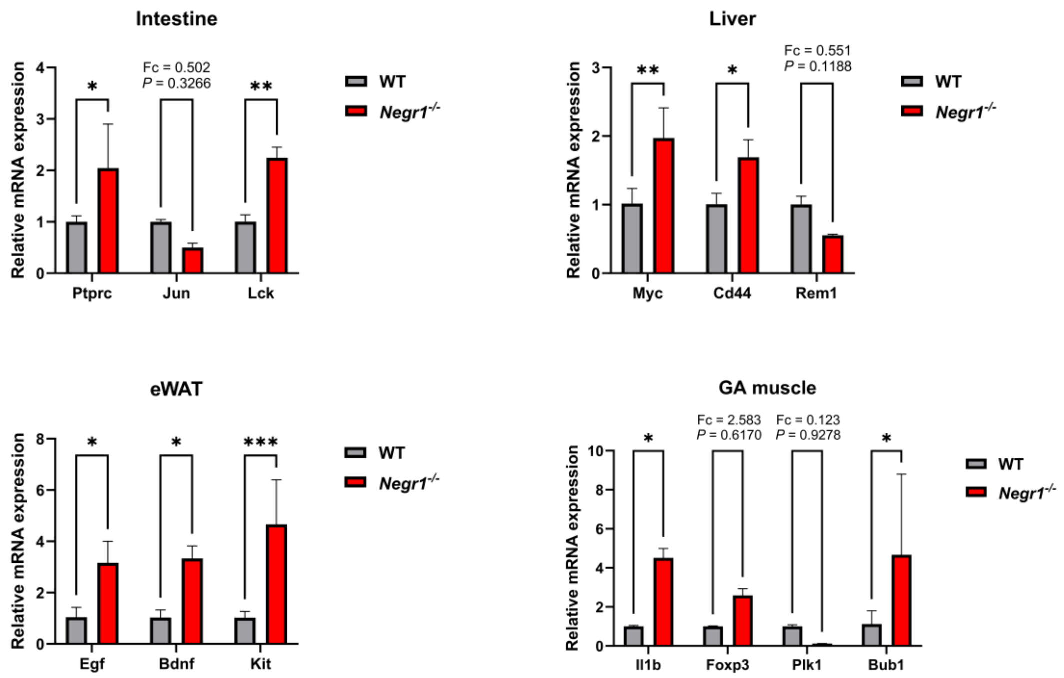

B

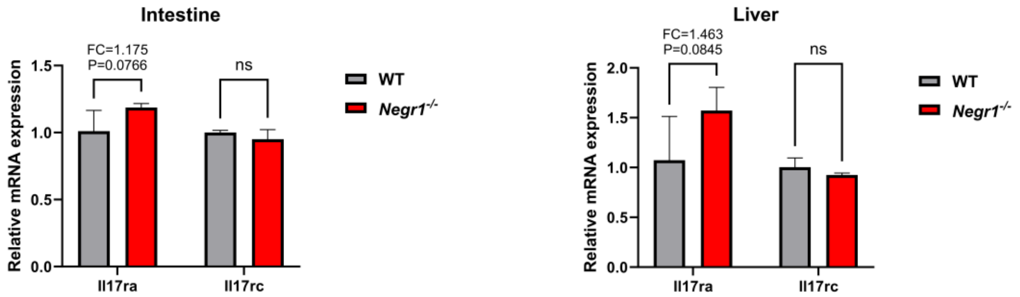

C

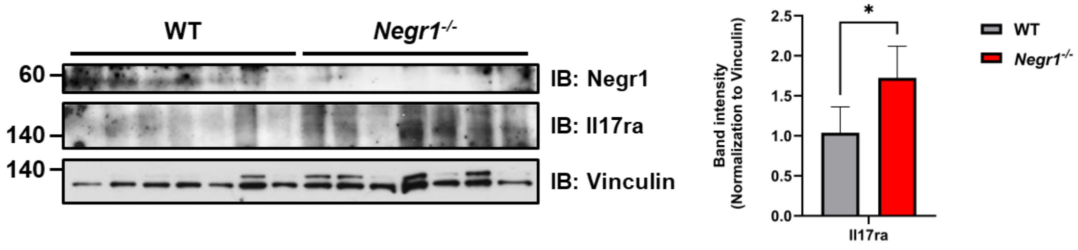

Supplementary Figure S2. (A) Validation of differential gene expression in WT and *Negr1*$^{-/-}$ mice by quantitative RT-PCR (qRT-PCR) for selected hub genes showing >2-fold changes in RNA-seq data for each tissue. Gene abbreviations: *Ptprc*, protein tyrosine phosphatase receptor type C; *Lck*, lymphocyte-specific protein tyrosine kinase; *Rem1*, RAD and GEM–related GTP-binding protein 1; *Myc*, MYC proto-oncogene; *Egf*, epidermal growth factor; *Bdnf*, brain-derived neurotrophic factor; *Kit*, KIT proto-oncogene receptor tyrosine kinase; *Foxp3*, forkhead box P3; *Bub1*, budding uninhibited by benzimidazoles 1. (B) mRNA expression levels of *Il17ra* and *Il17rc* in the intestine and liver, quantified by qRT-PCR. (C) IL-17A protein expression levels in the intestine of WT and *Negr1*$^{-/-}$ mice, assessed by immunoblotting. Band intensities were quantified using ImageJ software. Error bars represent ± SD. *, $p < 0.05$; **, $p < 0.01$; ***, $p < 0.001$.
