# Supplementary material for: Peripheral gene dysregulation in Negr1-deficient mice: insights into possible links with affective behavior
Source: Front Mol Neurosci. 2025 Jul 8;18:1602201. doi: 10.3389/fnmol.2025.1602201 (PMC12279845; doi:10.3389/fnmol.2025.1602201)
Supplement: Supplementary Data Sheet 3 — Colon length measurements of 16-week-old mice. [file Data_Sheet_3.pdf]

Supplementary Material

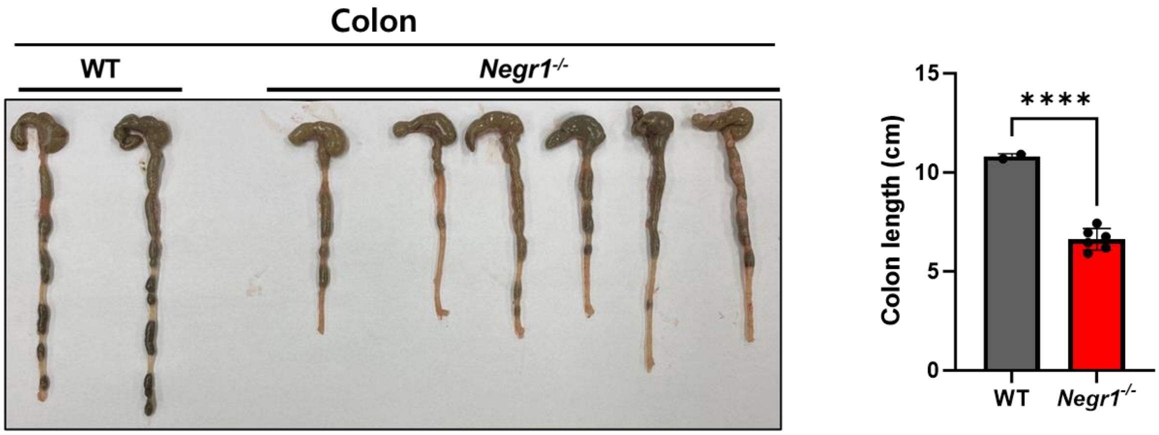

Supplementary Figure S3. Measurement of colon length of 16-week-old WT and *Negr1*$^{-/-}$ mice. Error bars represent ± SD. ****, $p < 0.0001$.
